# Supplementary figures and images for: Continuous mutation of SARS-CoV-2 during migration via three routes at the beginning of the pandemic
Source: PeerJ. 2022 Mar 30;10:e12681. doi: 10.7717/peerj.12681 (PMC8976469; doi:10.7717/peerj.12681)

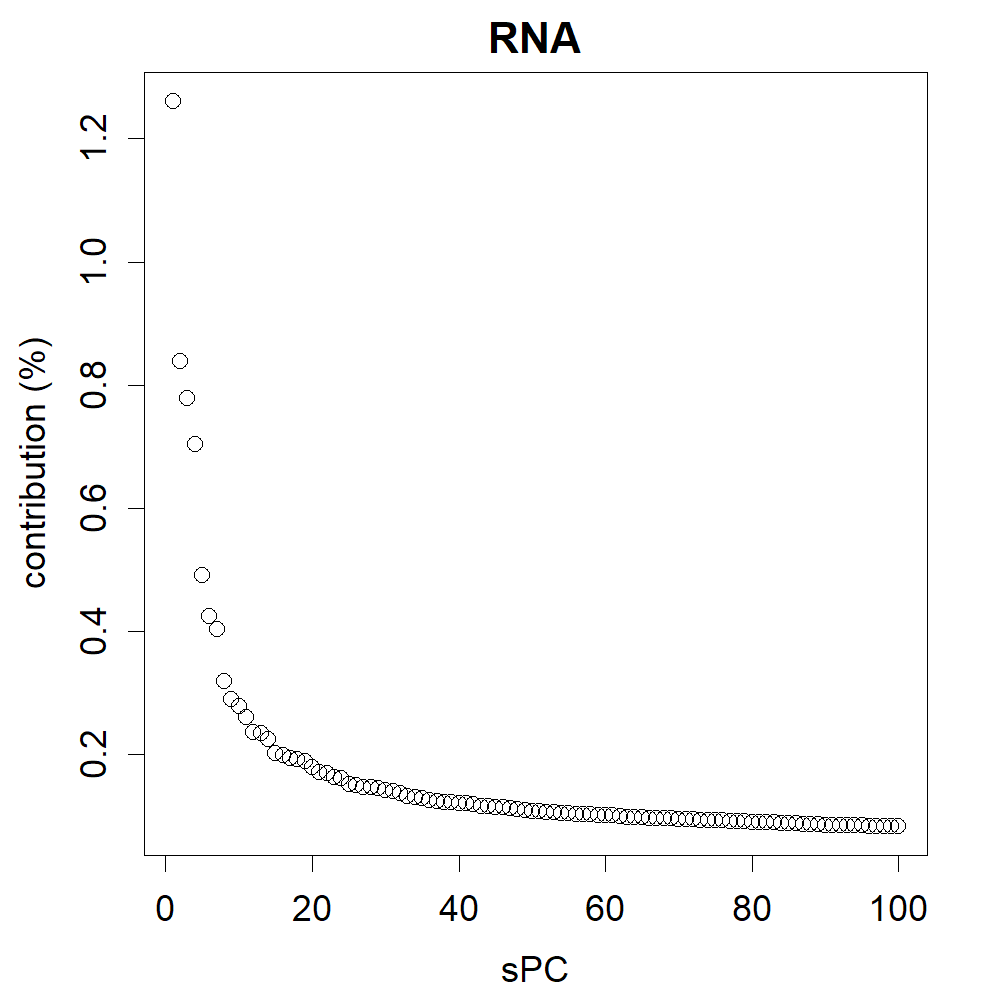

Supplement: Supplemental Information 1 — The contributions represent the extent to which each axis of the PC covers the differences between the data. In this case, PC1 is particularly large, and this is the axis that primarily represents the adaptation of the virus to humans as a host. [file peerj-10-12681-s001.png]

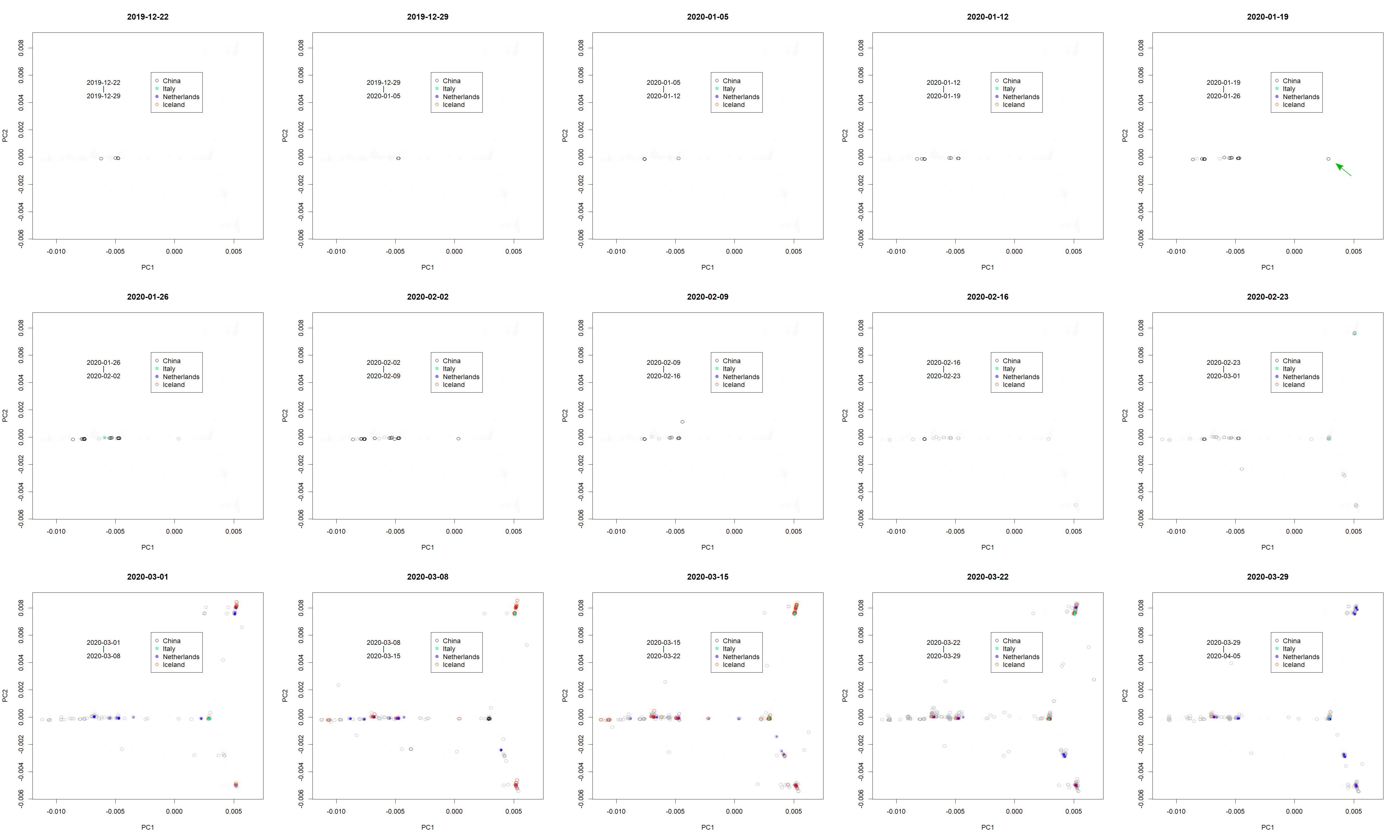

Supplement: Supplemental Information 2 — The formation of the routes became clear in the PCs. [file peerj-10-12681-s002.png]

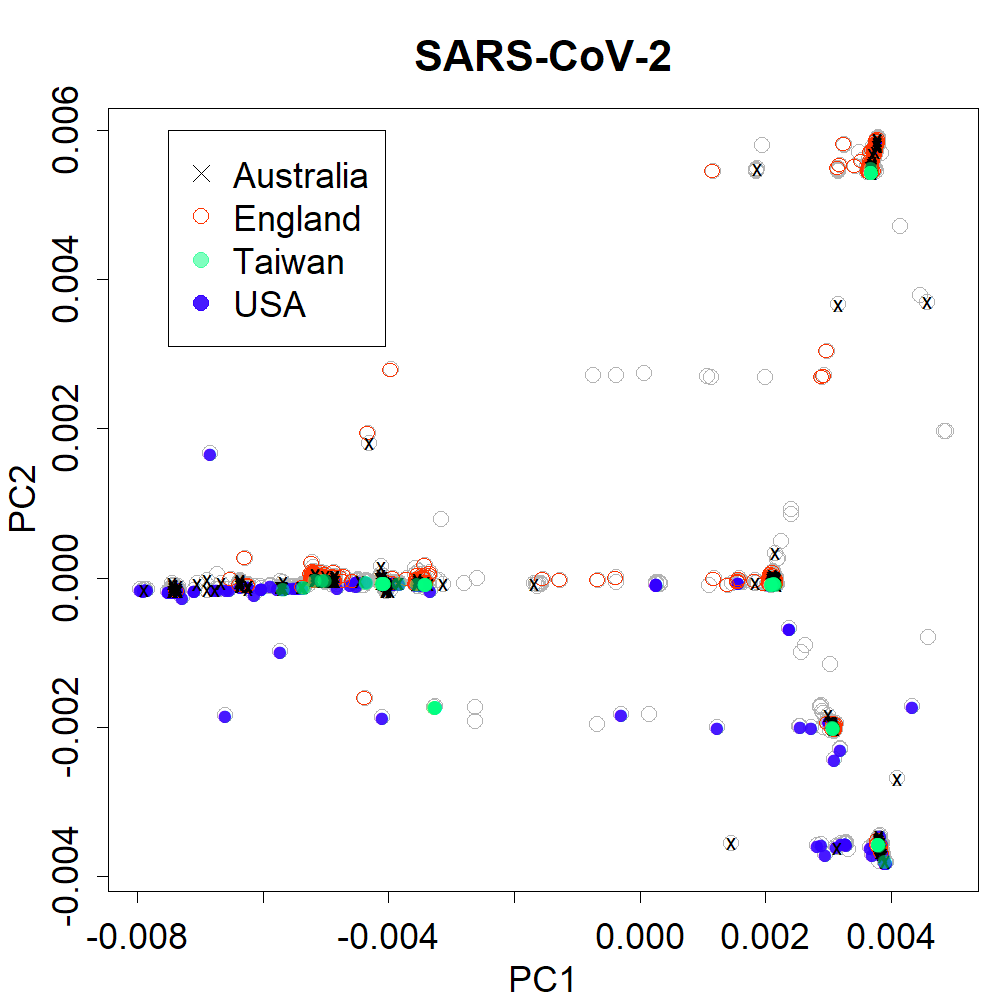

Supplement: Supplemental Information 3 — Those countries showed different patterns from Fig. 2A, showing uniqueness among each country. [file peerj-10-12681-s003.png]

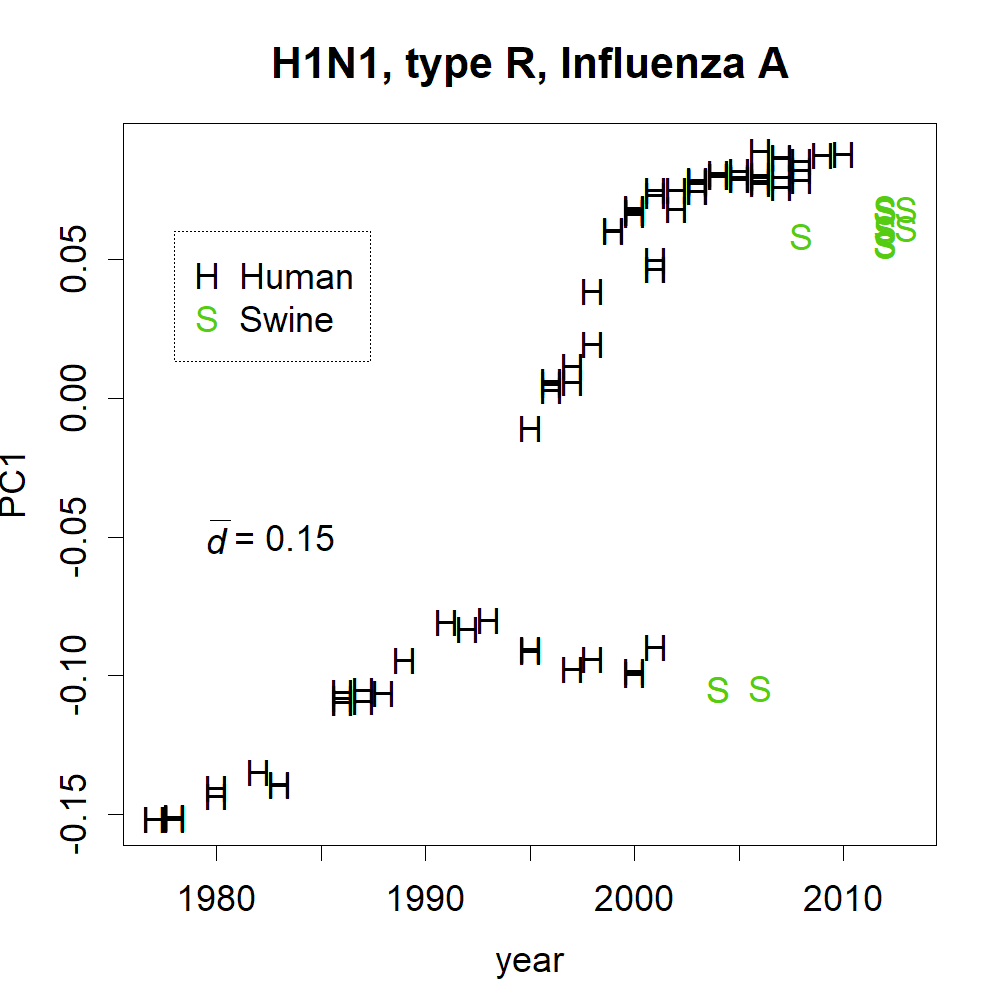

Supplement: Supplemental Information 4 — Changes in haemagglutinin are shown. [file peerj-10-12681-s004.png]
